# Effects of charge-modifying mutations in histone H2A α3-domain on nucleosome stability assessed by single-pair FRET and MD simulations

**DOI:** 10.1038/s41598-017-13416-x

**Published:** 2017-10-16

**Authors:** Kathrin Lehmann, Ruihan Zhang, Nathalie Schwarz, Alexander Gansen, Norbert Mücke, Jörg Langowski, Katalin Toth

**Affiliations:** 1 Division Biophysics of Macromolecules, German Cancer Research Center, Heidelberg, D-69120 Germany; 2 Present Address: Key laboratory of medicinal chemistry for natural resources, Ministry of Education, Yunnan University, Kunming, Yunnan 650091 China

## Abstract

Nucleosomes are important for chromatin compaction and gene regulation; their integrity depends crucially on the structural properties of the histone tails. Recent all-atom molecular dynamics simulations revealed that removal of the N-terminal tails of histone H3, known to destabilize nucleosomes, causes a rearrangement of two arginines of histone H2A, namely R81 and R88 by altering the electrostatic environment of the H2A α3 domain. Whether this rearrangement is the cause or the effect of decreased stability, is unclear. Here, we emulate the altered electrostatic environment that was found after H3 tail clipping through charge-modifying mutations to decouple its impact on intranucleosomal interactions from that of the histone tails. Förster resonance energy transfer experiments on recombinant nucleosomes and all-atom molecular dynamics simulations reveal a compensatory role of those amino acids in nucleosome stability. The simulations indicate a weakened interface between H2A-H2B dimers and the (H3-H4)_2_ tetramer, as well as between dimers and DNA. These findings agree with the experimental observations of position and charge dependent decreased nucleosome stability induced by the introduced mutations. This work highlights the importance of the H2A α3 domain and suggests allosteric effects between this domain and the outer DNA gyre as well as the H3 N-terminal tail.

## Introduction

The nucleosome, the basic unit of chromatin compaction, is central to gene regulation^1,2^. Two copies of each of the four histone proteins (H2A, H2B, H3 and H4) constitute the nucleosome core particle, around which approximately 150 bp of DNA are wrapped^3,4^. *In vivo*, nucleosomes modulate gene accessibility through a shift between their open and closed conformation and by changing their position on the DNA. The protruding, intrinsically disordered histone tails are important for the structural state of the nucleosome, and posttranslational modifications of these tails play a crucial role in gene regulation. Clipping of the protruding N-terminal domain of H3 *in vitro* unveiled that the H3 tail participates in intranucleosomal interactions by restricting breathing motion and compacting the nucleosome^5^. Hence, the H3 N-terminal seems to be fundamental for nucleosome stability^5–7^. Mechanistic information on the role of the histone tails in intranucleosomal interaction is still rare, though.

To understand the impact of H3 tail removal on nucleosome structure our laboratory recently performed molecular dynamics (MD) simulations based on the crystal structure 1KX5 of the nucleosome core particle^8^. Those analyses suggest an active role of two histone arginines within the H2A α3 domain in structural alterations. Arginines are highly flexible, positively charged amino acids, and important for histone-DNA interactions. In our simulations of intact nucleosomes, R81 of H2A interacts with H3 Q55/K56 and H2A G105/V107. Those interactions appeared to be broken after H3 tail removal, and new hydrogen bonds were formed between R81 and the nucleosomal DNA^8^. We postulated that the changed interaction is a result of a variation in the electrostatic potential at the H2A α3 domain induced by H3 tail clipping. Thus, the electrostatic potential at the H2A α3 domain may be a determining factor for nucleosome stability. Here we test this hypothesis by introducing charge-modifying mutations that alter the electrostatic potential at the H2A α3 domain without clipping of the H3 tail. Thus, we designed two sets of mutated, recombinant *Xenopus laevis* H2A histones. In the first set the positively charged arginine(s) are exchanged with neutrally charged alanine(s), while the second type incorporates a negative charge by exchanging arginine with glutamic acid.

To unravel the functional role of these arginines during nucleosome disassembly, we assessed the overall stability of mononucleosomes reconstituted on the Widom 601 DNA sequence^9^. *In vitro*, nucleosome disassembly can be forced by increasing salt concentrations and followed by Förster resonance energy transfer (FRET) spectroscopy. Distance-dependent energy transfer was measured between two fluorophores that are attached to different sites of the nucleosome. Typical Förster radii R_0_ of the dyes were around 5 nm, ideal for distance measurements within nucleosomes. Salt-induced destabilization and FRET have been widely used to analyze possible intermediate states in nucleosome compaction^10–15^. To reveal details of the disassembly process, we designed various nucleosome constructs, which are either labeled at two positions on the DNA or at one of the histone proteins and a suitable position on the DNA. With the help of similar, DNA labeled constructs the impact of tailless H3 histone on the nucleosome stability was elucidated^5^.

By comparing the H2A α3 mutants with the wild type, we could characterize the role of long distance interactions and the formation of intermediate states within the nucleosome. A state, with higher FRET, which was already observed in^16,17^ and is assumed to be connected with histone octamer opening, was promoted during nucleosome disassembly in the H2A α3 mutants. We conclude that these mutations weaken the interactions between H2A-H2B dimer and (H3-H4)_2_ tetramer and thereby lead to a decrease in nucleosome stability.

## Results

There is strong evidence that chromatin stability depends on intranucleosomal electrostatic interactions^18–20^. Previous molecular dynamics (MD) simulations on wild type and H3 tail-less nucleosomes suggested that the electrostatic potential of the H2A α3 domain could play an important role for nucleosome stability^8^. To test this hypothesis, we introduced site-specific point mutations at positions 81 and 88 in this domain, which change its electrostatic potential. We designed a total of six recombinant H2A mutants, where arginine was replaced with alanine (RA) or glutamic acid (RE) either at position 81 (R81A, R81E), at position 88 (R88A, R88E) or at both loci simultaneously (double mutants R81A/R88A, R81E/R88E).

**Figure 1:**
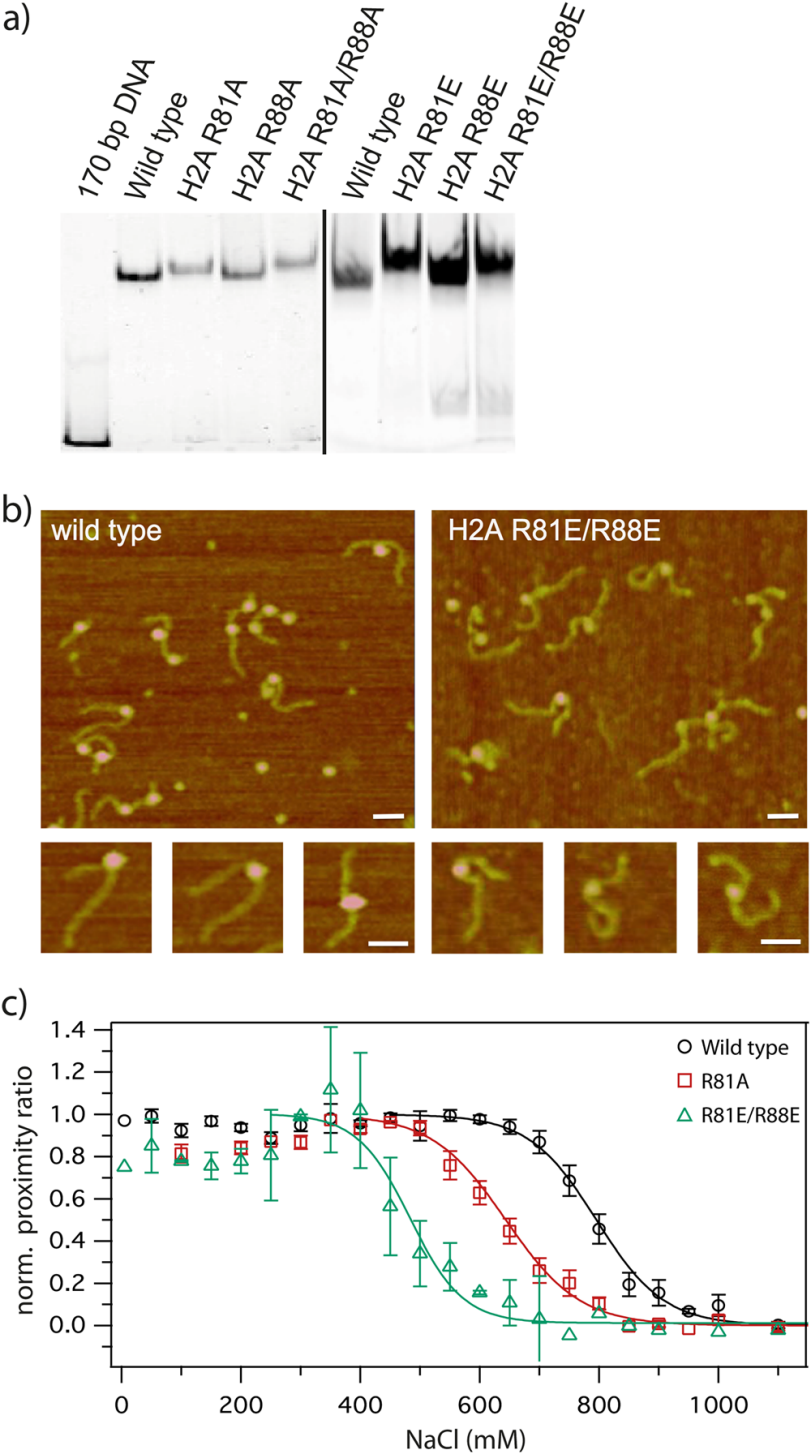
Comparison between wild type and mutated nucleosomes with different fluorescent and non-fluorescent based methods. (**a**) Electrophoretic mobility shift assay with reconstituted nucleosomes analyzed on native 6% PAAG-Gel. Successfully reconstituted nucleosomes bearing mutations in H2A and wild type nucleosomes show decreased electrophoretic mobility in comparison to free DNA. Small differences in electrophoretic mobility between the mutated nucleosomes are detectable suggesting changes of the hydrodynamic radius. (**b**) Representative AFM pictures of wild type and H2A R81E/R88E nucleosomes reconstituted on a 660 bp DNA containing Widom 601 sequence in the middle of the fragment. Scale bar = 50 nm. (**c**) Normalized average proximity ratio as a function of NaCl concentration of three independent replicates. Charge-modifying mutations reduced the overall stability of nucleosomes. Black: wild type, Red: H2A R81A mutant, Green: H2A R81E/R88E mutant, Symbols: measured values and standard error of the mean, Lines: sigmoidal fit.

Success of nucleosome reconstitution was analyzed by electrophoretic mobility shift assay (EMSA). For all reconstituted nucleosomes, wild type and H2A mutated nucleosomes, a significant gel shift was observed, indicating successful nucleosome reconstitution (Fig. 1a). Moreover, the small differences in gel mobility among the different nucleosome types might be caused by altered nucleosome conformations that lead to variations in hydrodynamic radius and gel mobility. Notably, all different nucleosome types show one sharp band, which gives evidence of preferred, sequence-dependent nucleosome positioning.

Additionally, we used Atomic force microscopy (AFM) to assess the influence of H2A mutations on nucleosome integrity as exemplified by comparison between H2A R81E/R88E and wild type. AFM is a powerful tool for analyzing nucleosome integrity^21–23^. Here, we used a 660 bp DNA fragment containing centrally the Widom positioning sequence, to analyze the formation of mono-, di- and trinucleosomes. A qualitative comparison revealed that both wild type and mutated nucleosomes are able to form mono-, di- and trinucleosomes, but occurrence of di- and trinucleosomes on mica surface was substantially diminished in H2A R81E/R88E nucleosomes (Fig. 1b). Both wild type and mutated mononucleosomes are mostly found in the central 601 Widom sequence, suggesting a loss of octamers on the flanking weaker positioning DNA sequence upon deposition. Furthermore, a reduction of the DNA length between free DNA (229 ± 16 nm, N = 82) and traceable DNA in wild type nucleosomes (188 ± 15 nm, N = 112) was observed and is in good agreement with earlier findings^23^. This reduction is proportional to the DNA length wrapped around the octamer. H2A R81E/R88E mutated mononucleosomes show a significantly smaller reduction of the DNA length (205 ± 19 nm, N = 80). Both, differences in number of deposited intact nucleosomes and in traceable DNA length between wild type and mutated nucleosomes, indicate changes of nucleosome integrity upon H2A mutation. Under the chosen deposition conditions, the nucleosome structure is influenced by electrostatic interactions between the negatively charged DNA and the positively charged poly-L-lysine surface as well as interactions between the proteins and the DNA. Nevertheless, we can consider the AFM results as a first hint towards differences in nucleosome stability caused by the introduced mutations as wild type and mutated nucleosomes were exposed to identical surface conditions. Further ensemble and single particle FRET (spFRET) measurements were performed in solution to analyze nucleosome stability and architecture in more detail.

### Microplate-scanning FRET measurements reveal a destabilizing effect of charge-modifying mutations

To analyze the influence of the introduced mutations on nucleosome disassembly, we measured distance changes between two positions on the outer DNA gyre (donor at +41 bp and acceptor at −53 bp from the middle of the DNA sequence, I_β_I_α_; see also Supplementary Figure S1A) using microplate-scanning FRET. Nucleosome concentration was set to 300 pM and samples were incubated for 1 h at salt concentrations from 5 – 1200 mM NaCl. The salt-dependent normalized proximity ratio (P) can be described by a sigmoidal function, whose point of half decay (c_(1/2)_-value) is taken as a measure for nucleosome stability (Fig. 1c). These bulk FRET experiments show that all H2A mutations destabilize the nucleosome, indicated by a lower c_(1/2)_-value (Table 1 c_(1/2)_-value ± SE).

**Table 1:** NaCl concentrations c_(1/2)_-values and standard errors at the inflection point of bulk FRET measured nucleosome disassembly.

Nucleosome	c_(1/2)_-value
WT	800 ± 29 mM
R88A	668 ± 23 mM
R81A	632 ± 23 mM
R81A/R88A	568 ± 20 mM
R88E	455 ± 19 mM
R81E	468 ± 15 mM
R81E/R88E	452 ± 15 mM

The observed stability decrease depends on the charge of the introduced amino acid: the effect is more prominent for all RE-mutants than for the respective RA-mutants (RA < RE). For the RA-mutations also the position of the amino acid is important for the induced destabilization (R88A < R81A < R81A/R88A). RE-mutants do not show this position dependence, but exhibit a much stronger increase of the initial P prior to the sigmoidal decrease than the other constructs (Fig. 1c). This increase of bulk P prior to the decrease is thought to represent the initial step of nucleosome disassembly, i.e. opening of the dimer:tetramer interface^16^. The change in curve shape may result from structural changes caused by the introduced mutations. This was further analyzed by spFRET experiments and all-atom MD simulations.

### spFRET suggests that charge-modifying mutations promote formation of disassembly intermediates

**Figure 2:**
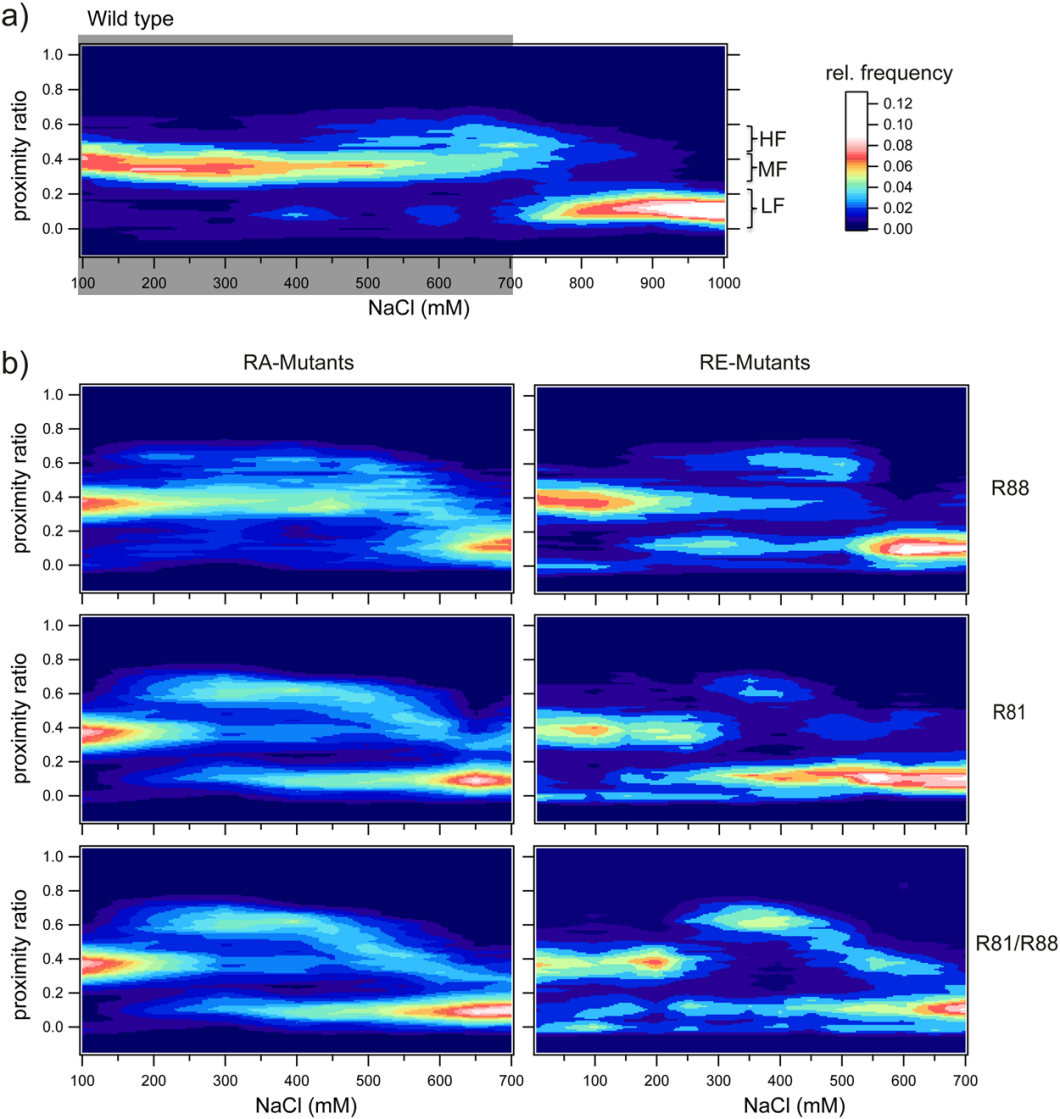
Contour plots of the proximity ratio distribution from single molecule measurements as a function of NaCl concentration for (**a**) wild type and (**b**) mutated I_β_I_α_ nucleosomes. Highlighted in gray: NaCl concentration range where mutated nucleosomes were measured (100 – 700 mM NaCl). The color scale represents the relative frequency. Wild type and mutated nucleosomes exhibit three distinct subpopulations with average proximity ratios of P = 0.39 for mid-FRET (MF), P = 0.64 for high-FRET (HF) state and P = 0.12 for low-FRET (LF) state. Comparison between wild type and mutated nucleosomes reveals two major differences which depend on charge (RA < RE) and position (R88 < R81 < R81/R88) of the introduced mutation: 1) HF is more prominent in the mutants and 2) the P distribution shows higher heterogeneity in the mutants.

I_β_I_α_ nucleosomes were used for spFRET measurements to analyze their structural heterogeneity upon salt-induced disassembly. The spFRET histograms of salt series (8 – 15 different salt concentrations in steps of 50 – 100 mM NaCl, examples will be presented and discussed later) were assembled to generate contour plots displaying the change of the P distribution as a function of the salt concentration (Fig. 2). The color scale of the contour plot refers to the frequency of the measured proximity ratio. This kind of representation facilitates the observation of transitions between different nucleosomal states during the salt dependent nucleosome disassembly and highlights the striking influences of the different mutations.

**Figure 3:**
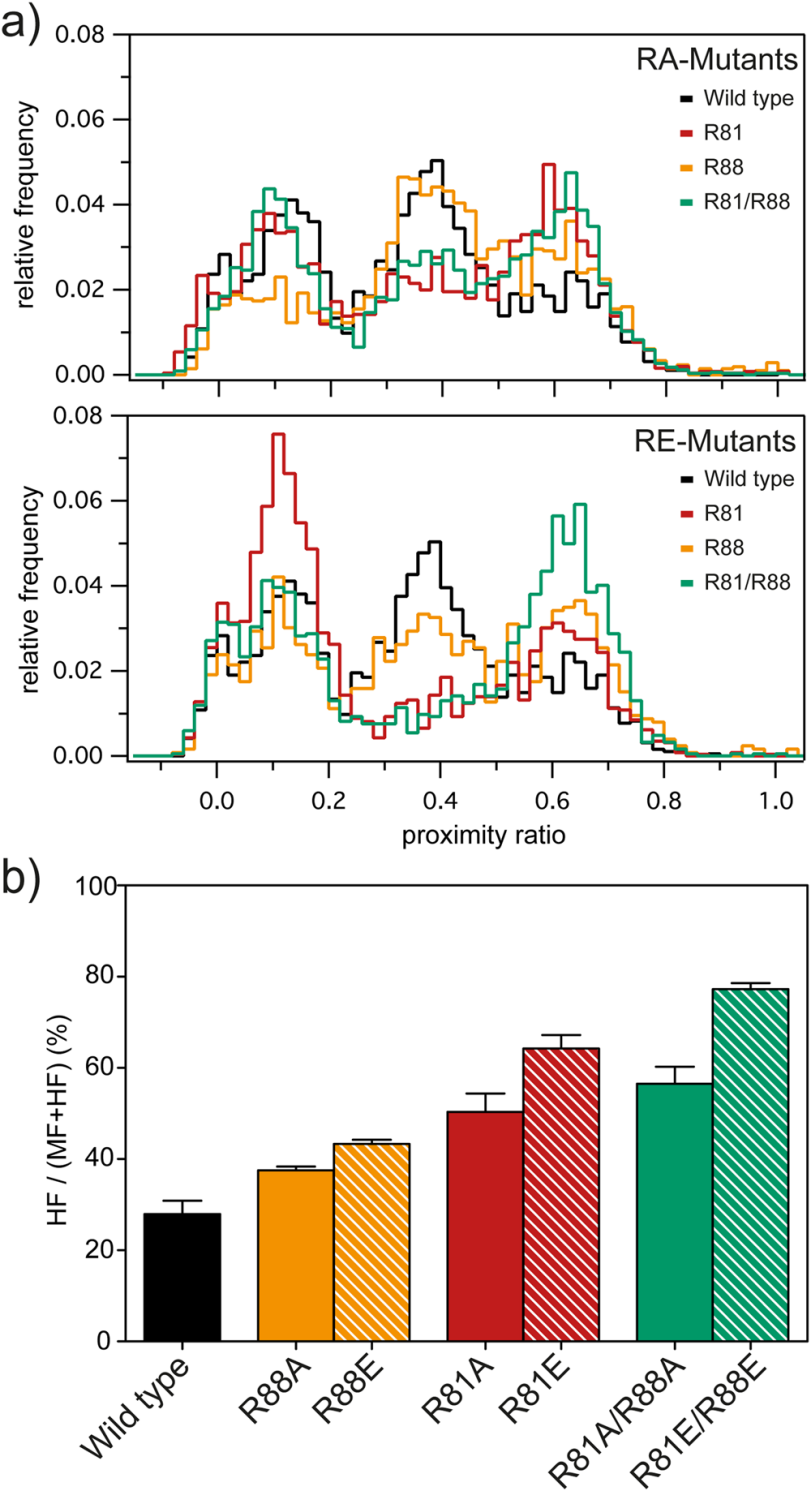
Mutations of R81 and R88 promote formation of disassembly intermediates in I_β_I_α_ nucleosomes. (**a**) Normalized spFRET histograms for wild type and RA-mutants (top), and for wild type and RE-mutants (bottom) at 400 mM NaCl. From each histogram the relative proportion of each of the three subpopulations (LF, MF, HF) was calculated from the areas multi Gaussian fit. (**b**) Percentage of partially disassembled nucleosomes (HF) relative to the sum of intact plus partially disassembled nucleosomes (MF + HF), elucidating correlations between enhancement of the HF state and decrease in nucleosome stability provoked by charge-modifying mutations in the H2A α3 domain. Averaged proportions and standard error of the mean were calculated from three independent replicas.

As described in^17^ wild type nucleosomes usually exhibit three distinct states with different proximity ratios (Supplementary Figure S2 for a schematic representation of nucleosomal states). At low salt (up to 600 mM NaCl) most wild type nucleosomes are found in a population around P ~ 0.39 (mid-FRET (MF) state), which represents the initial nucleosome conformation (Fig. 2a). At higher salt concentrations (600 – 800 mM NaCl) we observe the formation of a high-FRET (HF) state with an average P = 0.64. This can be explained by modification of the nucleosome structure or its partial disassembly bringing the two fluorophores closer together. The third population – the low-FRET (LF) state around P = 0.12 – can be detected at high salt concentrations (> 750 mM NaCl) and represents the open nucleosome or free DNA. The H2A-mutated nucleosomes feature populations with identical average P values (Fig. 2b), but comparison of the contour plots reveals two salient differences: 1) The HF species is more prominent in all mutants and 2) the P distribution is more heterogeneous in the mutated samples which can be seen by coexistence of different states in a broad region of NaCl concentration (300 – 600 mM). Figure 2b reveals that the extent of both effects depends on charge and position of the introduced mutation and complies with the trend observed in ensemble FRET measurements. Exchange of positively charged arginine with neutral alanine at a given position has less effect than the corresponding exchange with negatively charged glutamic acid (RA < RE). Furthermore, we note that the position of the mutation also influences the amount of the HF state as follows: R88 < R81 < R81/R88. The observed transition into the HF state is most striking at intermediate NaCl concentrations, as demonstrated for NaCl = 400 mM in Fig. 3a. Fitting the P distributions with the sum of three Gaussians, we estimated the fractions of each of the three subpopulations (LF, MF, HF) and calculated the percentage of partially disassembled nucleosomes (HF) relative to the remaining intact and partially disassembled nucleosomes (MF + HF) (Fig. 3b). This comparison indicates that the shift towards the high-FRET state depends on two factors: 1) charge/electrostatic potential (RA < RE) and 2) position (R88 < R81 < R81/R88) of the introduced mutation. The shift towards the HF state hints towards a weakened dimer:tetramer interaction, which might lead to a decreased energy barrier for dimer loss in the mutants. Taking together, single particle and ensemble FRET measurements confirmed that the enhancement of the HF state correlates with the decrease in stability of the mutated nucleosomes, demonstrating that the electrostatic potential at the H2A α3 domain directly influences nucleosome stability.

### Charge-modifying mutations alter the sequence of nucleosome disassembly steps

**Figure 4:**
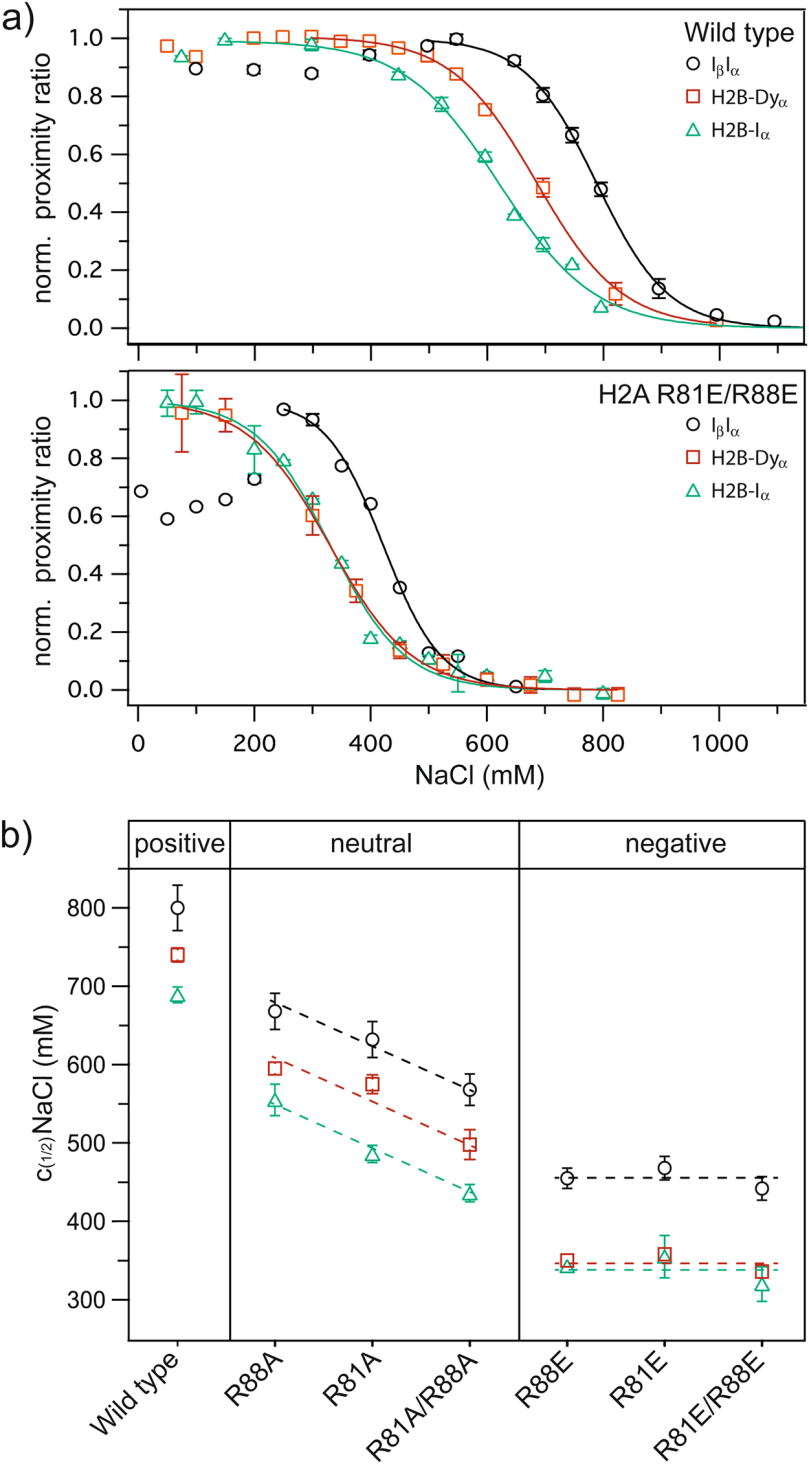
Salt-induced nucleosome disassembly monitored by various labeling strategies in microplate-scanning FRET experiments. (**a**) Representative representation of normalized average proximity ratio as a function of NaCl concentration for wild type (top) and mutated (bottom) nucleosomes with different labeling strategies. Black circles: I_β_I_α_ nucleosomes, Red squares: H2B-I_α_ nucleosomes, Green triangles: H2B-Dy_α_ nucleosomes, Lines: Sigmoidal fit. (**b**) Averaged c_(1/2)_-values and standard error of the mean (calculated from three independent replicas) for all constructs, revealing a decrease in overall stability of nucleosomes and changes of the nucleosome opening sequence with mutations. RE-mutants are less stable than the respective RA-mutants and show an altered nucleosome opening sequence. The effect of RA-mutations depends on the position of the introduced amino acid (R88A < R81A < R81A/R88A), but the nucleosome opening pathway is comparable to wild type nucleosomes.

For further analysis of the process we employed two labeling strategies suited to report on the state of the H2A-H2B heterodimer during nucleosome disassembly^16^ (Supplementary Figure S1B and C). 1) H2B-I_α_: donor on histone H2B and acceptor on the outer DNA turn, near the dimer (position −53 bp) and 2) H2B-Dy_α_: donor on histone H2B and acceptor on the inner DNA turn in vicinity to the dyad axis, near the (H3-H4)_2_ tetramer (position −15 bp). Together these constructs offer an indirect measure of the spatial arrangement of the H2A-H2B dimers with respect to the (H3-H4)_2_ tetramer, without the need for labeling H3 or H4 itself^16^. Previously, a comparison of these constructs for wt nucleosomes allowed us to propose a multi-step sequence of nucleosome disassembly and revealed the existence of a previously unknown intermediate state^16^. The sequential opening of wild type nucleosomes starts with opening of the DNA linker arms^17^, followed by opening of the dimer:tetramer interface and is completed by dimer eviction. The two-step opening of the dimer:tetramer interface and dimer eviction are clearly distinguishable through a significant difference of the c_(1/2)_-values between the two constructs H2B-Dy_α_ and H2B-I_α_^16^ (Fig. 4a, top). Here we used ensemble FRET experiments on H2B-I_α_ and H2B-Dy_α_ to probe the influence of the various H2A mutations on the disassembly pathway (Fig. 4a and b). The data confirm the destabilizing effect of the mutations, which again depends on position and charge of the introduced amino acid. Interestingly, the sequence of the nucleosome opening steps is not influenced upon RA-mutation, but appears to be altered upon RE-mutation, where the internal opening coincides with dimer dissociation (Fig. 4b). This finding suggests that nucleosome opening is facilitated due to weakened dimer:tetramer interaction, which consequently decreases the energy barrier for the dimer loss, in all the mutants. In RE-mutants a facilitated dimer eviction, leading to the comparable c_(1/2)_-values of both of the dimer constructs (H2B-I_α_ and H2B-Dy_α_), might be caused by decreased dimer:DNA interactions. We used all-atom MD simulations to investigate this assumption.

### Mutations alter the hydrogen bond environment of residues 81 and 88

To approach the mechanism of the reduced nucleosome stability in respect to 1) charge/electrostatic potential (RA < RE) and 2) position (R88 < R81 < R81R88) of the introduced mutations all-atom MD simulations were performed. Both mutations do not only change the charge state, to neutral (RA) or to negative (RE), but they also shorten the length of the side chains. To evaluate how these changes influence the local interaction network, we calculated the contact probability between H2A residues 81/88 and their surrounding residues.

**Figure 5:**
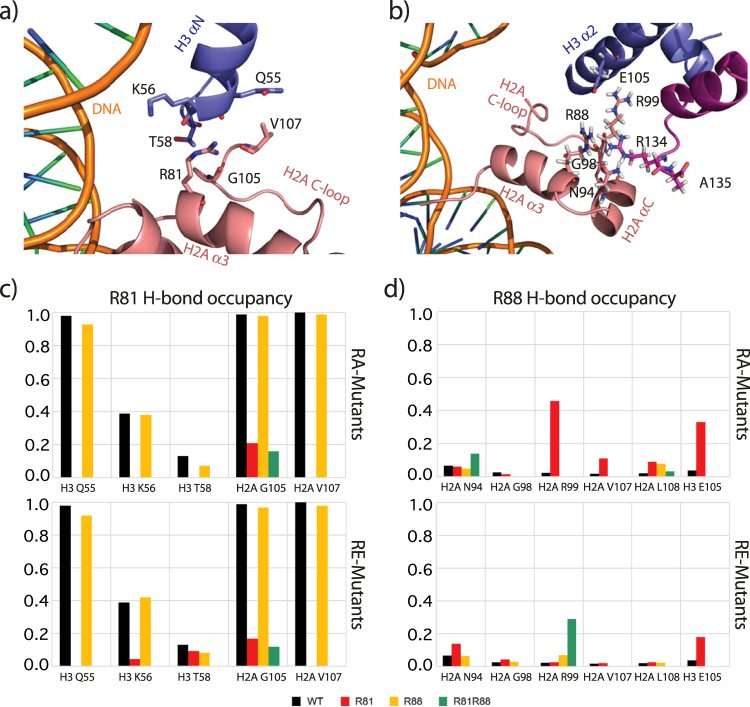
Local environment and hydrogen bond occupancy for H2A residues R81 (**a**) and R88 (**b**) in the crystal structure (PDB ID: 1KX5). Hydrogen bond occupancy for residues 81 (**c**) and 88 (**d**) after mutation. Black: wild type, Red: R81 mutated, Yellow: R88 mutated, Green: R81 and R88 mutated. Data were calculated from the 20 – 150 ns trajectory. Calculation was performed separately for both copies of H2A. (Only the data of copy 2 are shown here, data of copy 1 are shown in Supplementary Figure S3). Hydrogen bond occupancy of R88 increases upon R81 mutation, which indicates a compensatory role of R88. Residues R81 instead remain unchanged upon R88 mutation.

The simulations show that H2A R81 and R88 play different roles in maintaining nucleosome stability. As shown in Fig. 5a and b, R81 forms several stable hydrogen bonds with the surrounding residues, such as H3 Q55/K56 and H2A G105/V107. Thus, H2A R81 is centrally important for stabilizing the H3 αN helix and the H2A C-loop. In both H2A copies (Fig. 5 and Supplementary Figure S3), RA and RE mutations at position 81 abolish almost all contacts between R81 and the surrounding residues (H3 Q55/K56, H2A V107), and only a weak contact with H2A G105 is maintained (Fig. 5c). Interestingly, although having an opposite charge, the R81E mutation has less effect on the interaction pattern of R81 than the R81A mutation. This suggests that the size and hydrogen-bonding capacity of R81 may be more important for its short-range interaction than the positive charge.

R88, on the other hand, has very few direct polar interactions with the environment, except for weak contacts with H2A N94, G98 and V100 (Fig. 5b). Located in a less crowded environment, R88 lacks explicit and stable contacts with surrounding residues. The hydrogen bond occupancy is already very low in the wild type nucleosome (Fig. 5d); therefore, the influence of mutations is less significant. However, R88 is also important for maintaining the intact structure of the H2A C-loop (N89 – V100) and the C-tail (above T101). Notably, when R81 is mutated, R88 forms a new contact with H3 E105. This observation is in agreement with our previous study^8^, where R88 changes its orientation from the H2A C-loop to H3 E105 after H3 tail truncation. H3 E105 is located on the largest folded domain in H3, the helix α2. The hydrogen bond between H2A R88 and H3 E105 might help to compensate the destabilization induced by the R81A mutation. Thus, the influence of the position (R88 < R81 < R81R88) of the introduced mutation can be confirmed, whereas an influence of the net charge of the electrostatic potential (RA < RE) at the H2A α3 helix on the hydrogen bond network was not observed on the MD simulation times scale.

### Mutations alter the distance between both DNA arms

**Figure 6:**
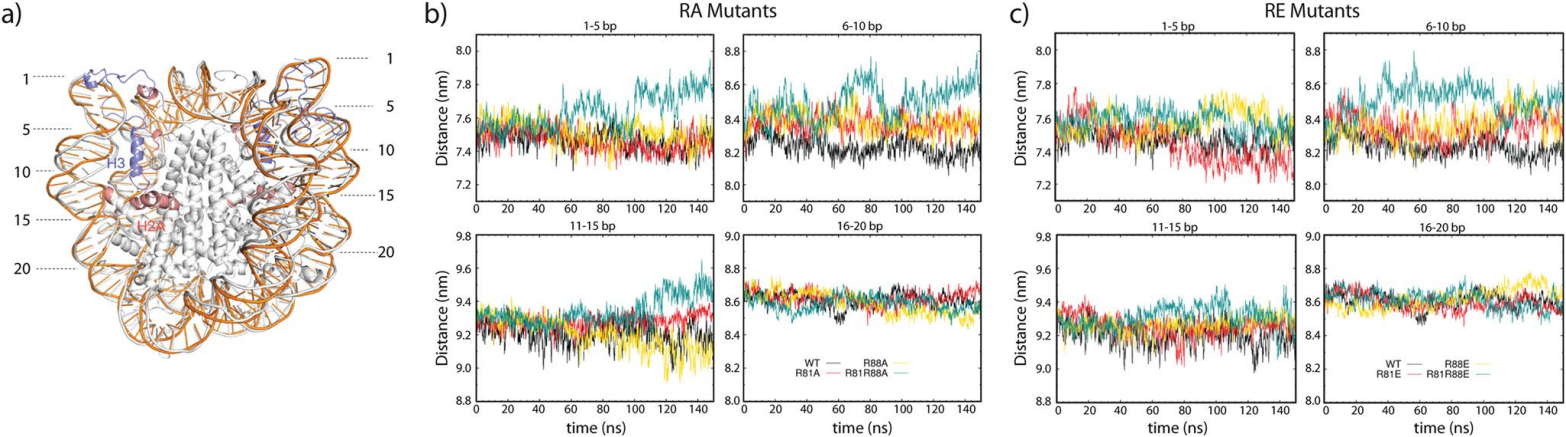
Distance between the two DNA arms. (**a**) Representation of considered DNA arms in the crystal structure (1KX5). The first 20 bp of DNA are labeled. Snapshots of WT (white) and R81 A/R88A (orange) at 80 ns were superimposed to show the increased distance between the DNA segments 6–15 bp away from the DNA ends in the mutated systems. (**b**) and (**c**) Distance between two DNA arms in RA and RE mutants. The center of mass was used for distance calculation. The running average of every 200 ps is plotted. Black: wild type, Red: R81 mutated, Yellow: R88 mutated, Green: R81 and R88 mutated.

In our MD simulations, only enhanced dimer fluctuation and expansion of DNA arms was observed, but without large conformational changes of the nucleosome such as DNA unwrapping. Nevertheless, by computing distances between the two DNA arms at entry/exit site we analyzed the influence of the H2A mutations on the wrapping of the nucleosomal DNA. Therefore, four segments of 5 bp each were classified (1–5 bp, 6–10 bp, 11–15 bp and 16–20 bp) and the distance between each segment and its counterpart on the opposite side of the nucleosome was calculated (Fig. 6). Although the simulation timescale (150 ns) is far below the millisecond time scale of partial DNA unwrapping^24^, it indicates that mutation of H2A R81 and R88 may increase the extent of DNA fluctuations. Compared to the wt, the distances between base pairs 6–10 are increased in the mutants, suggesting decreased histone-DNA interaction. For this segment both the double mutations (R81A/R88A, R81E/R88E) caused larger expansion of DNA arms compared to the single mutation (R81A, R88A, R81E and R88E); but an influence of the net charge of the electrostatic potential (RA < RE) at the H2A α3 helix on these fluctuation was not observed.

**Figure 7:**
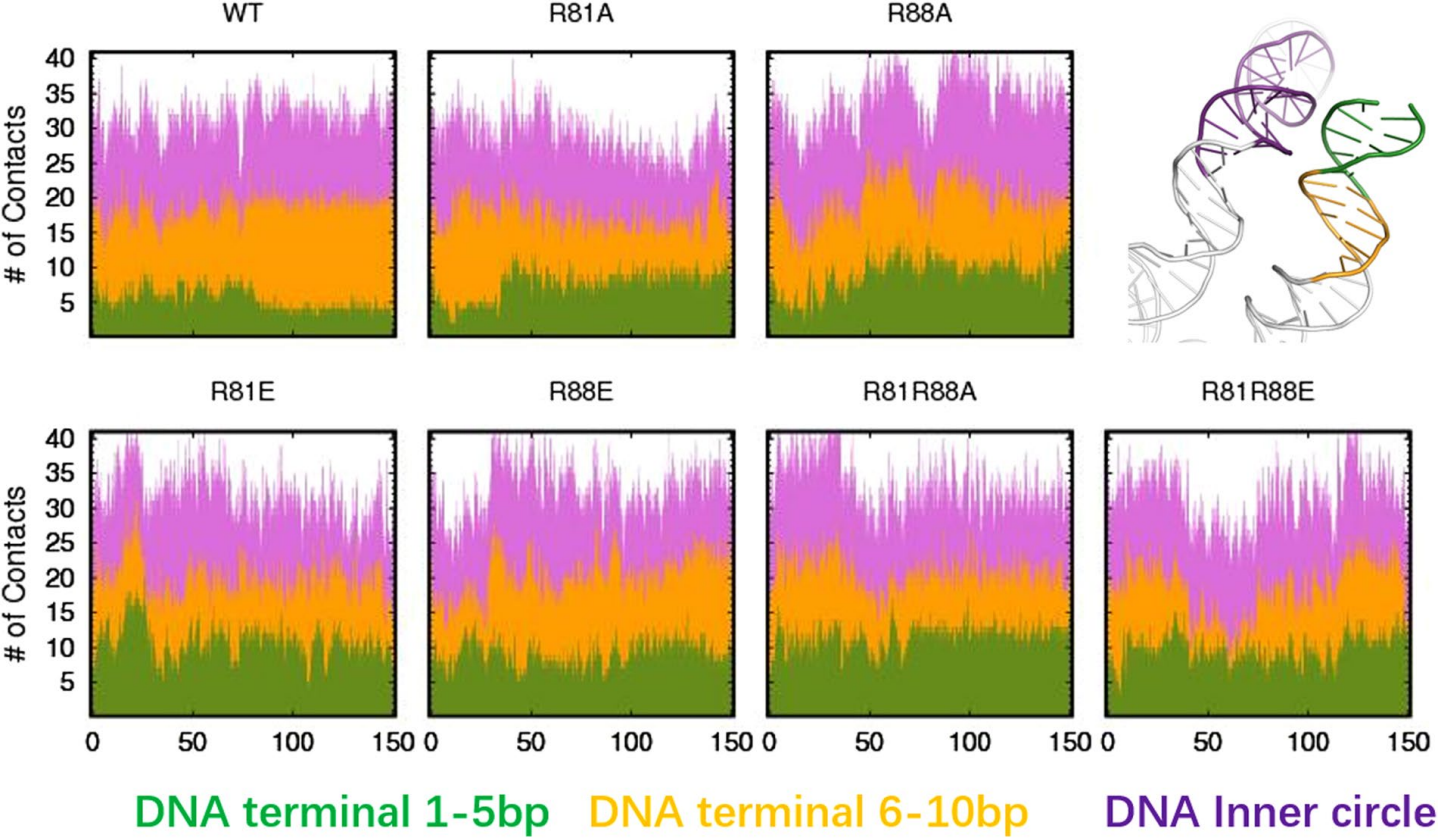
Number of contacts between the H3 tail and different segments of DNA. The Y axis shows the accumulated number of contacts. Different colors represent the contacts with different parts of the DNA. Green: H3 tail – DNA arm 1–5 bp. Yellow: H3 tail – DNA arm 6–10 bp. Purple: H3 tail – DNA inner gyre. Contact is defined as a distance between non-hydrogen atoms of less than 0.35 nm. The calculation was done for H3 tails in copy 2.

Interestingly, the very ends of the DNA arms (first 5 bp), which are supposed to be very flexible, are less affected than the 6–10 bp segment. This is related to the behavior of the histone H3 tails (1–26 amino acids), which may “hold” the first 5 bp of DNA in place to avoid DNA unwrapping. To verify this hypothesis, the number of contacts between H3 copy 2 and different segments of DNA was calculated. The H3 tail is attached to the first 10 bp of DNA arm and the inner gyre (see Fig. 6a). Comparing all simulated nucleosomes (Fig. 7), we found that although the total number of H3 tail-DNA contacts is not much different, the fraction of contacts between the H3 tail and the first 5 bp (displayed in green) of DNA is increased in all mutants. At the same time, the interactions between the H3 tail and the remaining part of the DNA, such as the inner gyre (displayed in purple) and base pairs 6–10 (displayed in orange) are decreased. This is particularly obvious for the R81A/R88A and R81E/R88E double mutants, where the contacts with base pairs 6–10 are mostly replaced by contacts with the first 5 bp. In summary, the H3 tail shifts towards the first 5 bp of DNA and prevents the DNA arms from “peeling off”, by sacrificing other interactions with DNA.

## Discussion

The N-terminal histone tails have long been recognized as target sites for posttranslational modifications, but they also play a pivotal role in their unmodified form for controlling nucleosome integrity. Proteolytic removal of the H3 tail, for example, has been shown to decrease nucleosome stability^25^ and to increase transient unwrapping of the DNA ends^5^. Previous simulations have revealed structural fluctuations in the histone core upon H3-tail truncation, which might be caused by an altered orientation of two arginines (R81 and R88) in the H2A α3 domain^8^. Consequently, the interaction network of R81 and R88 appeared to be altered and a more negative electrostatic potential of this domain was predicted. Based on these simulations the interesting question arose, whether the electrostatic potential at the H2A α3 domain has a direct influence on the overall stability of nucleosomes. Here, we addressed this open question by investigating the effect of charge-modifying mutations at these sites using all-atom MD simulations and FRET experiments.

Ensemble FRET indicates a destabilization of nucleosomes upon mutation in H2A α3 domain, which depends on the position (R88 < R81 < R81/R88) and the charge (RA < RE) of the mutations. The spFRET data revealed that the destabilization correlates with an increase of an intermediate species (HF) that is transiently formed during nucleosome disassembly. We assume that formation of the HF state involves weakening of the dimer:tetramer interface. Here, we show that we generated a series of mutations to further weaken the dimer:tetramer interface, which triggers a successive overrepresentation of the HF state. The conversion from MF (intact nucleosomes) into HF (modified nucleosomes) depends on the position (R88 < R81 < R81/R88) and charge (RA < RE) of the introduced mutations, which is in accordance with the ensemble FRET results. Importantly, besides the increase in HF we also detected a change in the sequence of nucleosome disassembly steps upon RE mutation: coinciding c_(1/2)_-values of H2B-I_α_ and H2B-Dy_α_ reveal that opening of the dimer:tetramer interface and dimer eviction occur at similar ionic strength. Recently, evidence was found that nucleosome disassembly occurs asymmetrically, starting with eviction of one dimer and followed by eviction of the second dimer^26^. Mutations in the H2A α3 domain may facilitate the eviction of the first dimer, thus accelerate internal nucleosome dynamics and consequent disassembly. Both, decreased nucleosome stability and increase in HF population can be explained by all-atom MD simulations, which revealed that H2A R81 and R88 play different roles in maintaining intact nucleosome structure. In wild type nucleosomes, hydrogen bonds between R81 and H3Q55/K56 stabilize the electrostatic contacts between DNA and H3 αN helix, which is extremely important for holding the entry-exit site. Even though R88 has less direct interactions with neighboring residues, it seems to be crucial for maintaining the electrostatic environment and thus defining the stability of the H2A C-loop and C-tail.

In addition, R88 may also play a compensatory role. In our simulations, upon R81 mutation R88 acts as a stabilizer of dimer:tetramer interaction, by forming a salt bridge with H3 E105. Exactly the same effect had already been observed in MD simulations after H3 histone tail-truncation^8^, suggesting allosteric effects between H2 α3 domain and the H3 tail. Calculation of the contact probability between H2A R81 and R88 and their neighboring residues confirmed a cross-talk between R81 and R88. Notably, R81 mutations significantly change the contact network of R88, whereas R88 mutation has only little influence on R81. MD simulations and FRET experiments thus concur with the conclusion that R81 is more important for the stability of the histone core. But, both arginines seem to be important for maintaining the disassembly sequence. Furthermore, MD simulation showed a larger structural change for the double mutants than the single mutants, however, no significant structural differences were observed between the MD simulations of the RA and RE mutations. This discrepancy between FRET measurements and MD simulations results is likely due to the different timescales of the two methods. The MD simulation time may be insufficiently small compared to the real structural alteration timescale of nucleosomes. Similar to our findings the importance of the local electrostatic potential in isolated subunits of nucleosomes has been reported recently^27^. They found by all atom MD simulations and fluorescence colocalization experiments that a charge-modifying point mutation at a strategic point (S68E) of the centromere protein A CENP-A disrupts chaperone binding.

Our H2A mutations seem to induce both local changes in the interaction network as well as allosteric changes causing not only H3-tail conformation changes but also DNA arm expansion. This finding is eminently important as it gives deeper insight into the pathways by which nucleosomes disassemble. The H2A histone protein, which has the highest genetic variability among all histone proteins, may play a crucial role in regulating nucleosome stability. FRET and ultracentrifugation experiments already unveiled that some of the H2A variants lead to an increased stability (e.g. MacroH2A^28^), whereas others may decrease nucleosome stability (e.g. H2A.Bbd^29^). Many of these H2A variants exhibit changes within the so-called docking domains, which consists of the H2A c-loop and the α3 domain analyzed here. Interestingly, R81 appears to be conserved in almost all H2A variants (except H2A.Bbd), whereas quite some variants show alterations in R88^30^. The position dependent differences in appearance of mutations within α3 domain confirm our hypothesized compensatory role of H2A R88. In addition, the respective mutations of H2A histone (R82A and R89A) in *Saccharomyces Cerevisiae* (SK1) did not result in growth defect under mitotic conditions (personal communication, Lóránt Székvölgyi, Dept of Biochemistry and Molecular Biology, University of Debrecen, Hungary). For position R82 in *Saccharomyces cerevisiae* the viability was confirmed in another study, but with impaired initiation of transcription, DNA replication and DNA repair^31^.

Single point mutations, changing the overall stability of the nucleosomes without affecting the general nucleosomal structure, as shown here, further emphasize the importance of the chemical composition of the nucleosome components. Hitherto, most of the analyzed mutations and posttranslational modifications had only small effects on the overall nucleosome stability^32,33^. In case of H3 K56Ac an increase in nucleosome breathing and a small effect on nucleosome remodeling was observed^33^. Our hydrogen bond analysis revealed that the stable interaction of H2A R81 with H3 K56 in wild type nucleosomes is diminished upon R81A/E mutation. Thus, our observations underline the importance of the H2A α3 domain and the significance of the dimer:tetramer interface for nucleosome integrity.

## Material and Methods

### Mutagenesis of histone H2A

Two sets of mutated, recombinant *Xenopus laevis* H2A histones were designed via overlap extension PCR. Each set comprises either a single amino acid exchange or the respective double mutation at position 81 and position 88.

The first set incorporates neutrally charged alanine(s) at the respective position(s), whereas the second type incorporates negatively charged glutamic acid.

The sequences of the required primers are shown in Supplementary Table S1. The mutated H2A sequences were cloned into the vector pet17b (Addgene) using the intrinsic restrictions sites for *NdeI* and *NotI*.

### Protein purification and labeling

Mutated H2A proteins were overexpressed, isolated and purified as described in^34^. Overexpression was performed in *E. coli* BL21(DE3) after induction with IPTG. 3 h after induction of protein expression proteins were isolated and purified. The three steps of the purification protocol^34^ are: 1) preparation of the isolated inclusion bodies, 2) size exclusion chromatography under denaturing conditions and 3) ion-exchange chromatography under denaturing conditions. After lyophilization proteins can be stored at −20 °C^34^.

All other recombinant wild type histones and H2BT112C, which was used for protein labeling, were purchased from Planet Protein (Colorado State University). Prior to octamer reconstitution histone H2BT112C^14^ was specifically labeled at the inserted cysteine with Alexa 488 Maleimide as previously described^16^. Labeling was performed under unfolding conditions (7 M guanidine hydrochloride, 20 mM Tris-HCl, 10 mM dithiothreitol, pH 7,5) with 10-fold excess of TCEP.

### Octamer reconstitution

Histone octamers were prepared by mixing equimolar concentrations of the core histones with 20% excess of H3 and H4 under unfolding conditions (7 M guanidine hydrochloride, 20 mM Tris-HCl, 10 mM dithiothreitol). Followed by overnight dialysis against refolding buffer (10 mM Tris-HCl, 0.1 mM EDTA, 5 mM β-mercaptoethanol; 2 M NaCl) at 4 °C in Slide-A-Lyser cassettes (MWCO 7000, Pierce). After size exclusion with FPLC (Superdex 200HR 10/10) all fractions were analyzed via Triton X-100/acetic acid/urea (TAU) gel analysis and fractions containing all four core histones in best proportion were selected for nucleosome reconstitution.

### DNA preparation and reconstitution of labeled mononucleosomes

DNA was prepared from the pGEM3z vector containing the Widom 601 positioning sequence^9^ via PCR with fluorescently labeled primers (IBA) and purified on Gen-Pak FAX HPLC (Waters). Alexa 488 (donor) and Alexa 594 (acceptor) were used as a dye pair for FRET-measurements. Fluorophores were attached to the DNA through amino-C6 linker.

The labeling efficiency of the DNA was determined to >95% by absorption spectroscopy and single molecule fluorescence with alternating laser excitation (ALEX). For single molecule FRET experiments protein labeling was purposefully set to 5–10% to minimize the presence of double donor labeled octamers.

Nucleosomes were reconstitution on 170 bp long DNAs containing the 601 Widom sequence^9^ via salt dialysis as described in^16^. DNA and octamers were mixed in a molar proportion between 1:1.7 and 1:2.2 in high salt (10 mM Tris-HCl, 0.1 mM EDTA, 2 M NaCl). Sample was transferred into mini dialyzing tubes (Pierce) and salt concentration was continuously decreased via dialysis (to 10 mM Tris-HCl, 0.1 mM EDTA, 5 mM NaCl). Quality of the reconstituted nucleosomes was analyzed by gel electrophoresis on either 6% polyacrylamide gels (60:1 acrylamide: bisacrylamide) in TBE buffer at pH = 7.5 at 10 V/cm or 2% agarose gels in 0.5 × TBE buffer at 10 V/cm. Only nucleosomes with less than 5% free DNA and fluorescence anisotropy of the attached dyes of less than 0.2 were used for experiments. Wild type and mutated nucleosomes can be stored at 4 °C for several weeks. As nucleosomes were reconstituted on the non-palindromic Widom 601 sequence, the two sides of the nucleosome where assigned with α- and β-side and the positions are counted from the middle of the fragment. Abbreviations for the three types of nucleosomes used for FRET measurements start with the position and side of the donor, followed by position and side of the acceptor (see also Supplementary Figure S1):

I_β_I_α_:          DNA labeled at +41 bp (Alexa 488) and −53 bp (Alexa 594)

H2B-I_α_:    H2B-T112C (Alexa 488) and DNA labeled at −53 bp (Alexa 594)

H2B-Dy_α_: H2B-T112C (Alexa 488) and DNA labeled at −15 bp (Alexa 594)

Accessible volume (AV) simulations were performed with the FPS toolkit to calculate dye position distributions^35^.

### AFM measurements

For AFM measurements nucleosome samples (c_Nuc_ = 1.5 nM in 10 mM Tris-HCl, pH 7.5, 0.1 mM EDTA, 15 mM NaCl) were deposited on a freshly cleaved mica pre-treated with 30 µl of an aqueous 4 mg/l poly-L-lysine solution (Sigma-Aldrich)^36^. After incubation for 1 min, the surface was gently washed with distilled water and dried with a constant stream of nitrogen. Images were recorded with a Nanoscope IIIa (Digital Instruments), software version 5.12r3, operated in “tapping mode” with silicon probes (type: PointProbe^®^ Plus, NCH, NanoAndMore GmbH). Contour lengths of free DNA were measured from one end of the molecule to the other end by tracing the backbone of the DNA with the program ImageJ^37^. DNA length of nucleosomes was measured similarly by drawing a curved line through the center of the histone-DNA complex.

Wild type and mutated nucleosomes used for AFM were reconstituted with 6 to 10-fold molar excess of octamer on a 660 bp DNA fragment. The DNA fragment was generated by enzymatic cleavage of pGem3z (Widom 601, Addgene) plasmids with the restriction enzyme *PvuII*.

### Microplate scanning FRET analysis

Bulk FRET was measured in a 384-well microplate on a Typhoon multimode imager as described in^38^. Nucleosomes were diluted to 300 pM and incubated for 60 min in 0.02 µm-filtered TE-Buffer at pH = 7.5 containing 5 – 1200 mM NaCl, 1 mM ascorbic acid (Sigma-Aldrich) and 0.01% Nonidet P40 (Roche Diagnostics). FRET was quantified by recording donor emission I_D_^Dex^ between 500 – 540 nm and acceptor emission I_A_^Dex^ between 595 – 625 nm after excitation at 488 nm. Direct acceptor excitation I_A_^Aex^ was recorded at 532 nm excitation and detected between 595 – 625 nm. Donor-only, acceptor-only, double labeled DNA fragments with no FRET, and the buffer solution were measured simultaneously as control samples.

The proximity ratio (P) was calculated from the recorded fluorescence intensities after correcting for background, crosstalk of Alexa 488 into the acceptor channel and direct excitation of the acceptor at 488 nm^38^.

1 $$P=\frac{{({I}_{A}^{{D}_{ex}})}_{corr}}{{({I}_{A}^{{D}_{ex}})}_{corr}+\,{({I}_{D}^{{D}_{ex}})}_{corr}}$$

Each experiment contained technical triplicates. For further analysis, normalized average proximity ratios and standard deviation were plotted against NaCl concentration and fitted with a sigmoidal function

2 $$P(X)=P(0)\times \frac{P(\infty )-P(0)}{1+{e}^{(\frac{{c}_{1/2}-x}{b})}}$$

in which X represents the salt concentration in mM. P(0) and P(∞) are maximum amplitude and offset of the fit curve, c_(1/2)_ is the inflection point of the curve and b the slope at the inflection point. If not stated otherwise independent triplicates were used to calculated average c_(1/2)_-value and standard error of the mean.

### Single-pair FRET experiments

The structural heterogeneity of labeled mononucleosomes was analyzed by single-pair FRET experiments in solution as described in^39^. The overall nucleosome concentration was adjusted to 300 pM by mixing 50 pM of labeled nucleosomes and 250 pM of unlabeled nucleosomes that were prepared with octamers of the same mutational state. Mononucleosomes were incubated in the same way as described in microplate-scanning FRET experiments. From the data stream, provided by a time-correlated-single-photon-counting board (TimeHarp200, PicoQuant), bursts were defined as a group of at least 50 photons with a mutual separation of less than 120 µs^38^.

For each individual burst a proximity ratio (P) was calculated as

3 $$P=\frac{{N}_{A}}{{N}_{A}+\,{N}_{D}}$$

Here, N_A/D_ represent the photon numbers detected in the donor and acceptor channel after correcting for background and donor crosstalk into the acceptor channel^38^.

From all calculated Ps probability distributions were built with a bin with of 0.02. Subpopulations with different average P were dissected with multi-Gaussian fits, where the relative areas underneath each peak were used to compute the fractions of intact (MF), partially disassembled (HF) and open nucleosomes/free DNA (LF)^39^. Average fraction size and standard error of the mean were calculated from independent triplicates.

The proximity ratio (P) follows the trend of the FRET efficiency (E) and can be taken as a measure for distance changes between the two fluorophores. P and E are related to each other through the setup dependent detection factor (γ-factor) for both the microplate scanning and spFRET

4 $$E=\frac{{N}_{A}}{{N}_{A}+\,\gamma \,{N}_{D}}=\frac{1}{1+\,{(\frac{R}{{R}_{0}})}^{6}}$$

5 $$P=\frac{\gamma \,}{\gamma -\,1\,+\,\frac{1}{{\rm{E}}}}=\frac{\gamma }{\gamma \,+\,{(\frac{R}{{R}_{0}})}^{6}}$$

where R_0_ is the Förster radius, R is the distance between the fluorophores and *γ* depends on the different detection efficiencies (η) and the quantum yields (ϕ) of the donor and acceptor fluorophores.

6 $$\gamma =\frac{{\eta }_{A}\,\times {\varphi }_{A}}{{\eta }_{D}\times {\varphi }_{D}}$$

### Molecular dynamics simulation

The simulations were based on the nucleosome crystal structure with complete histone tails and 147 bp DNA (PDB ID: 1KX5)^3^. The structure was solvated in a cubic box filled with explicit water molecules as described by the TIP3P model^40^. The minimum distance between the edge of the box and the solute atom was set to 1.0 nm. Na^+^ were added to neutralize the system charge, and then 200 mM NaCl were added to keep the salt concentration similar to the experimental conditions. To investigate the influence of H2A R81/R88 mutations, seven nucleosome models were constructed: wild type nucleosome (wt), nucleosome with a single RA mutation on R81 or R88 (R81A, R88A), double RA mutations on R81 and R88 (R81A/R88A), single RE mutation on R81 or R88 (R81E, R88E) and double RE mutations on R81 and R88 (R81E/R88E). Both histone copies contained the mutations. The definition of copy 1 and 2 of histones is shown in Supplementary Figure S4.

All simulations were performed with the Gromacs 4.6 program^41^ using the Amber99SB force field^42^. The cut-off for non-bonded interaction was 1.2 nm, and the long-range electrostatic interaction was treated with the particle-mesh Ewald (PME) method^43^. The LINCS algorithm was applied to constrain all bonds of hydrogen atoms^44^, so that the time step for integration was set to 2 fs. The initial structures underwent three steps of energy minimization: 1) in vacuum, 2) in solvent with nucleosome atoms fixed and 3) in solvent with all atoms free. The minimization stopped when the maximum force in the system dropped below 100 kJ∙mol^−1^∙nm^−1^. Then, the systems were heated to 300 K equilibrated during 200 ps at constant number of atoms, volume, and temperature (NVT), followed by 200 ps equilibration with constant number of atoms, pressure, and temperature (NPT) the pressure was set to around 1 bar. In both equilibration phases the nucleosome atoms were restrained. Then the folded tail conformation on the wt nucleosome was sampled by a 100 ns NPT MD simulation with all atoms free. Based on the snapshot at 100 ns, the mutated systems were constructed and subjected to 150 ns MD simulations (Supplementary Figure S4).

### Data Availability

The datasets generated and/or analyzed during the current study are available from the corresponding author on reasonable request.

## Electronic supplementary material

Supplementary Information
