# Supplementary material for: Effects of charge-modifying mutations in histone H2A α3-domain on nucleosome stability assessed by single-pair FRET and MD simulations
Source: Sci Rep. 2017 Oct 16;7:13303. doi: 10.1038/s41598-017-13416-x (PMC5643395; doi:10.1038/s41598-017-13416-x)
Supplement: Supplementary file 1 — Supplementary Information [file 41598_2017_13416_MOESM1_ESM.pdf]

Effects of charge-modifying mutations in histone H2A α3-domain on nucleosome stability assessed by single-pair FRET and MD simulations

Kathrin Lehmann[1+*], Ruihan Zhang[1+#], Nathalie Schwarz[1], Alexander Gansen[1], Norbert Mücke[1], Jörg Langowski[1†] and Katalin Toth[1*]

[1] Division Biophysics of Macromolecules, German Cancer Research Center, Heidelberg, D-69120 Germany

[#] Present address: Key laboratory of medicinal chemistry for natural resources, Ministry of Education, Yunnan University, Kunming, Yunnan 650091, China

* To whom correspondence should be addressed. Tel: + 49 - (0)6221 - 42 3394; Fax: + 49 - (0)6221 - 42 3391; Email: k.lehmann@dkfz.de, Tel: + 49 - (0)6221 - 42 3390; Fax: + 49 - (0)6221 - 42 3391; Email: kt@dkfz.de

[†] Deceased, 6th May 2017

[+]The authors wish it to be known that, in their opinion, the first two authors should be regarded as joint First Authors.

Supplementary information:

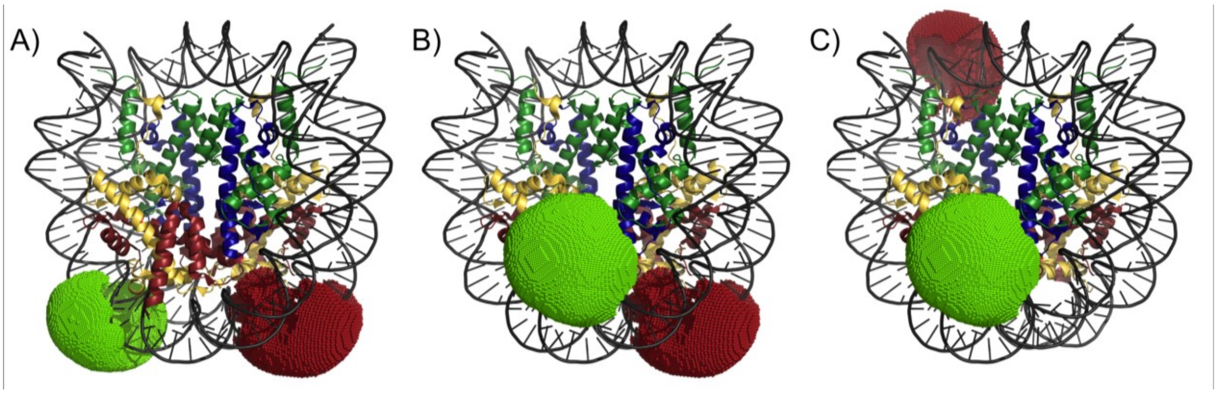

Figure S1: Representation of labeling positions. Nucleosomes crystal structure (3LZ1) and accessible fluorophore space for donor (light green) and acceptor (red). Histones are shown in blue (H3), forest green (H4), ruby (H2A) and yellow (H2B). A) $I_\beta I_\alpha$: Alexa488 at +41bp DNA, Alexa594 at -53 bp DNA, B) H2B-I_α: Alexa488 at H2B and Alexa 594 at -53 bp DNA, C) H2B-Dy_α: Alexa488 at H2B and Alexa 594 at -15 bp DNA.

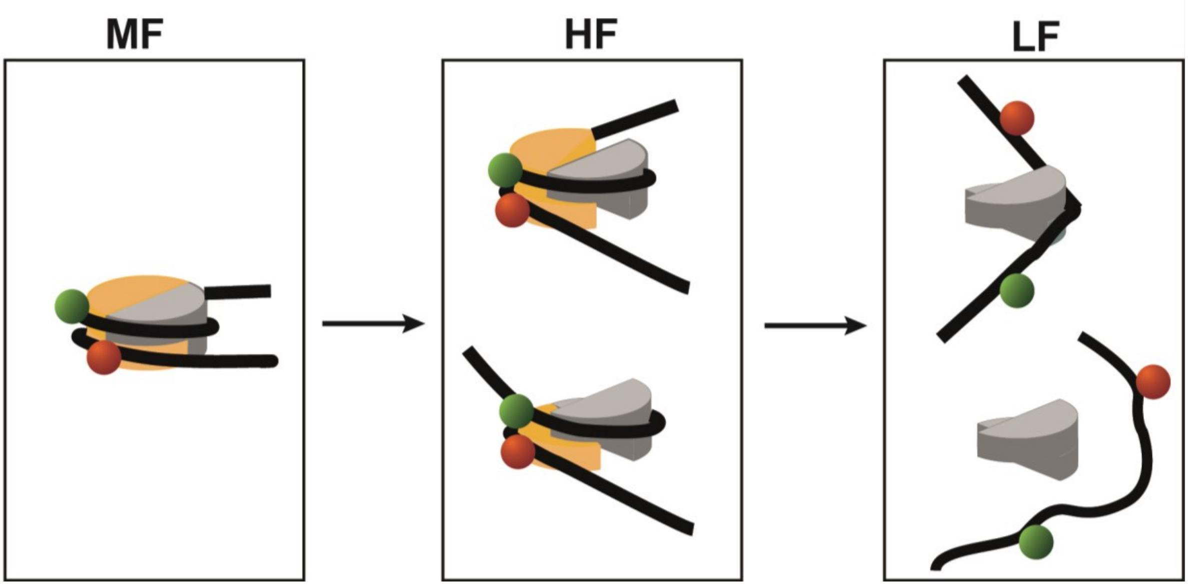

Figure S2: Nucleosomal states during disassembly. spFRET measurements with $I_\alpha I_\beta$ nucleosomes reveal three distinct states nucleosomal with P ~ 0.39 - mid-FRET (MF) state, P = 0.64 - high-FRET (HF) and P = 0.12 - low-FRET (LF) state. In the schematic both H2A-H2B Dimer is shown in yellow, $(H3\text{-}H4)_2$ tetramer in grey, DNA in black, donor in green and acceptor in red.

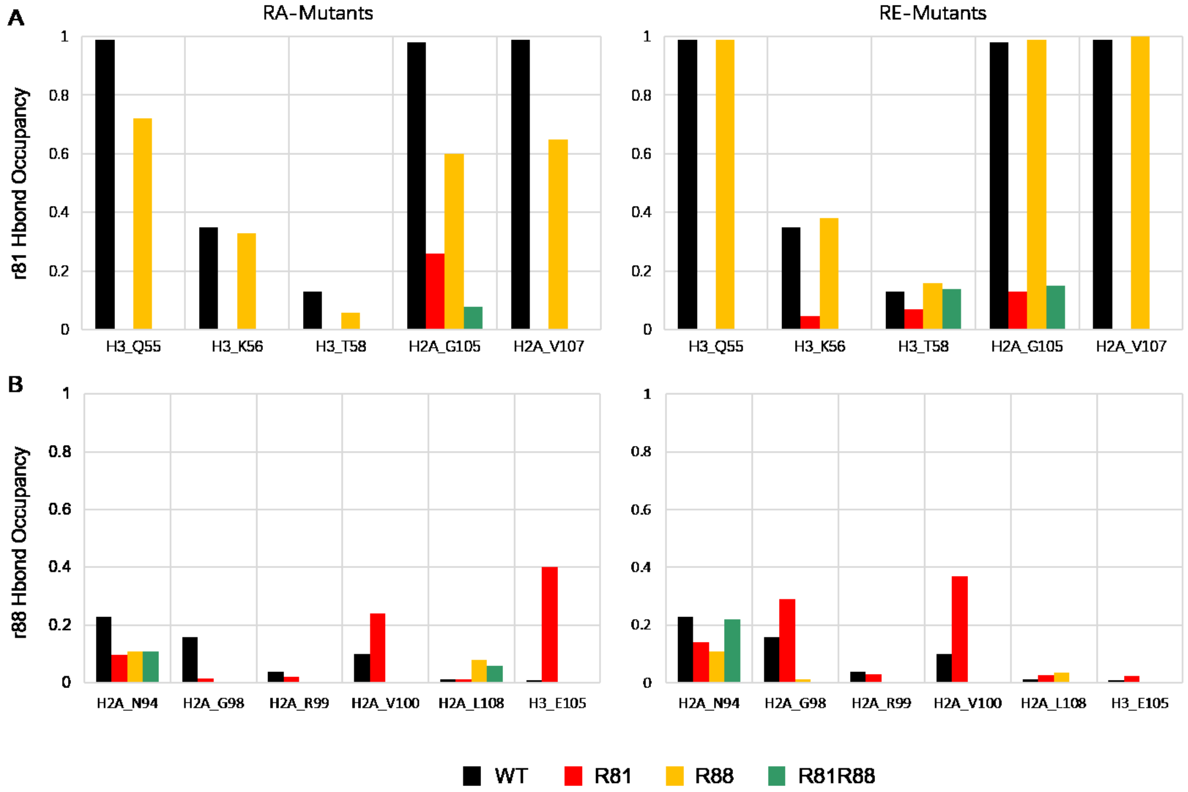

Figure S3 Hydrogen bond occupancy for H2A residue 81 and 88 in H2A copy 1. Hydrogen bond occupancy for residue 81 (A) and residue 88 (B) were calculated from the 20-150ns trajectory.

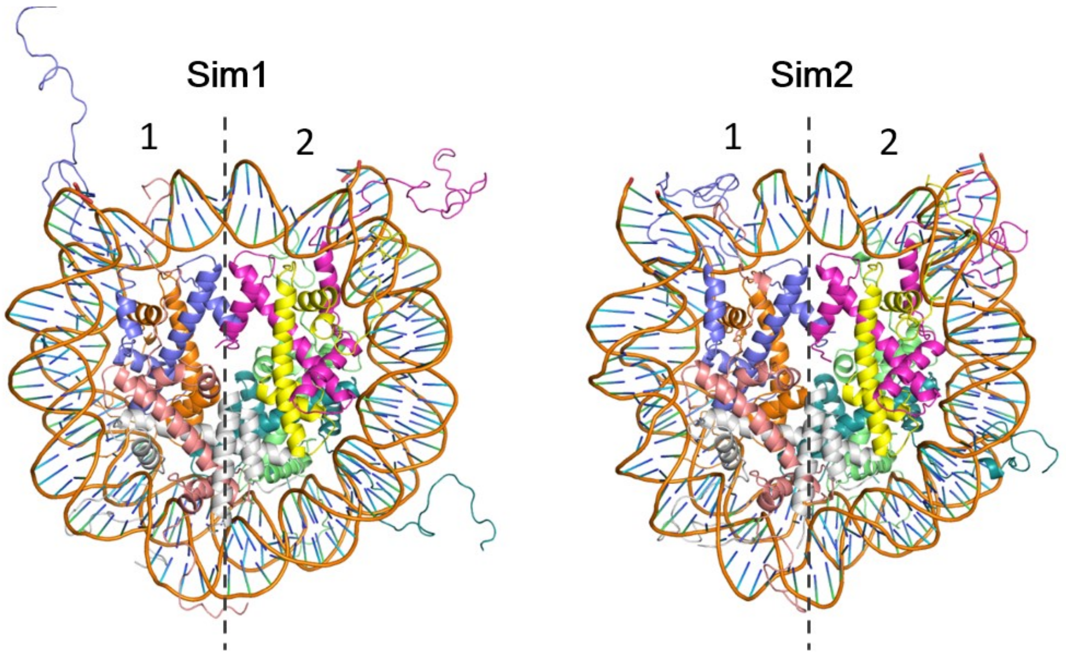

Figure S4 Initial structure of Sim1 and Sim2. Sim 1: 100ns NPT equilibration was performed on the wild type crystal structure (PDB ID: 1KX5). Sim 2: Starting from the snapshot of Sim 1 at 100ns, 150ns production run was performed for wild type and all mutated systems. H3: copy 1 purple blue and copy 2 magenta. H4: copy 1 orange and copy 2 yellow. H2A: copy 1 salmon and copy 2 light green. H2B: copy 1 white and copy 2 teal.

Tab S1: Primers for overlap extension PCR

Name	Sequence 5' → 3'
H2A Nde I forward	TATTATCATATGTCAGGAAGAGGCAAACAA
H2A Not I reverse	ATATAGCGGCCGCGTTTACTTGCTCTTGGCCGA
H2A R81A forward	GCAGGTGTGCGGGGATAATGC
H2A R81A reverse	GCATTATCCCCGCACACCTGC
H2A R88A forward	CTCATCGTTGGCCACAGCGAG
H2A R88A reverse	CTCGCTGTGGCCAACGATGAG
H2A R81E forward	GCATTATCCCCGAACACCTGC
H2A R81E reverse	GCAGGGTGTTCGGGGATAATGC
H2A R88E forward	CTCGCTGTGGAGAACGAGAG
H2A R88E reverse	CTATCGTTCTCCACAGCGAG
